# United States Private-Sector Physicians and Pharmaceutical Contract Research: A Qualitative Study

**DOI:** 10.1371/journal.pmed.1001271

**Published:** 2012-07-24

**Authors:** Jill A. Fisher, Corey A. Kalbaugh

**Affiliations:** 1 Center for Biomedical Ethics & Society, Vanderbilt University, Nashville, Tennessee, United States of America; 2 Department of Epidemiology, Gillings School of Global Public Health, University of North Carolina at Chapel Hill, Chapel Hill, North Carolina, United States of America; Yale University School of Medicine, United States of America

## Abstract

**Background:**

There have been dramatic increases over the past 20 years in the number of nonacademic, private-sector physicians who serve as principal investigators on US clinical trials sponsored by the pharmaceutical industry. However, there has been little research on the implications of these investigators' role in clinical investigation. Our objective was to study private-sector clinics involved in US pharmaceutical clinical trials to understand the contract research arrangements supporting drug development, and specifically how private-sector physicians engaged in contract research describe their professional identities.

**Methods and Findings:**

We conducted a qualitative study in 2003–2004 combining observation at 25 private-sector research organizations in the southwestern United States and 63 semi-structured interviews with physicians, research staff, and research participants at those clinics. We used grounded theory to analyze and interpret our data. The 11 private-sector physicians who participated in our study reported becoming principal investigators on industry clinical trials primarily because contract research provides an additional revenue stream. The physicians reported that they saw themselves as trial practitioners and as businesspeople rather than as scientists or researchers.

**Conclusions:**

Our findings suggest that in addition to having financial motivation to participate in contract research, these US private-sector physicians have a professional identity aligned with an industry-based approach to research ethics. The generalizability of these findings and whether they have changed in the intervening years should be addressed in future studies.

*Please see later in the article for the Editors' Summary.*

## Abstract 2

Jill Fisher and Corey Kalbaugh describe their findings from a qualitative research study evaluating the motivations of private-sector physicians conducting contract research for the pharmaceutical industry.

## Editors' Summary

**Background:**

Before a new drug can be used routinely by physicians, it must be investigated in clinical trials—studies that test the drug's safety and effectiveness in people. In the past, clinical trials were usually undertaken in academic medical centers (institutes where physicians provide clinical care, do research, and teach), but increasingly, clinical trials are being conducted in the private sector as part of a growing contract research system. In the US, for example, most clinical trials completed in the 1980s took place in academic medical centers, but nowadays, more than 70% of trials are conducted by nonacademic (community) physicians working under contract to pharmaceutical companies. The number of private-sector nonacademic physicians serving as principal investigators (PIs) for US clinical trials (the PI takes direct responsibility for completion of the trial) increased from 4,000 in 1990 to 20,250 in 2010, and research contracts for clinical trials are now worth more than USṩ11 billion annually.

**Why Was This Study Done?:**

To date, there has been little research on the implications of this change in the conduct of clinical trials. Academic PIs are often involved in both laboratory and clinical research and are therefore likely to identify closely with the science of trials. By contrast, nonacademic PIs may see clinical trials more as a business opportunity—pharmaceutical contract research is profitable to US physicians because they get paid for every step of the trial process. As a result, pharmaceutical companies may now have more control over clinical trial data and more opportunities to suppress negative data through selective publication of study results than previously. In this qualitative study, the researchers explore the outsourcing of clinical trials to private-sector research clinics through observations of, and in-depth interviews with, physicians and other research staff involved in the US clinical trials industry. A qualitative study collects non-quantitative data such as how physicians feel about doing contract research and about their responsibilities to their patients.

**What Did the Researchers Do and Find?:**

Between October 2003 and September 2004, the researchers observed the interactions between PIs, trial coordinators (individuals who undertake many of the trial activities such as blood collection), and trial participants at 25 US research organizations in the southwestern US and interviewed 63 informants (including 12 PIs) about the trials they were involved in and their reasons for becoming involved. The researchers found that private-sector physicians became PIs on industry-sponsored clinical trials primarily because contract research was financially lucrative. The physicians perceived their roles in terms of business rather than science and claimed that they offered something to the pharmaceutical industry that academics do not—the ability to carry out a diverse range of trials quickly and effectively, regardless of their medical specialty. Finally, the physicians saw their primary ethical responsibility as providing accurate data to the companies that hired them and did not explicitly refer to their ethical responsibility to trial participants. One possible reason for this shift in ethical concerns is the belief among private-sector physicians that pharmaceutical companies must be making scientifically and ethically sound decisions when designing trials because of the amount of money they invest in them.

**What Do These Findings Mean?:**

These findings suggest that private-sector physicians participate as PIs in pharmaceutical clinical trials primarily for financial reasons and see themselves as trial practitioners and businesspeople rather than as scientists. The accuracy of these findings is likely to be limited by the small number of PIs interviewed and by the time that has elapsed since the researchers collected their qualitative data. Moreover, these findings may not be generalizable to other regions of the US or to other countries. Nevertheless, they have potentially troubling implications for drug development. By hiring private-sector physicians who see themselves as involved more with the business than the science of contract research, pharmaceutical companies may be able to exert more control over the conduct of clinical trials and the publication of trial results than previously. Compared to the traditional investigatorinitiated system of clinical research, this new system of contract research means that clinical trials now lack the independence that is at the heart of best science practices, a development that casts doubt on the robustness of the knowledge being produced about the safety and effectiveness of new drugs.

**Additional Information:**

Please access these websites via the online version of this summary at http://dx.doi.org/10.1371/journal.pmed.1001271.

- The ClinicalTrials.gov website is a searchable register of federally and privately supported clinical trials in the US; it provides information about all aspects of clinical trials
- The US National Institutes of Health provides information about clinical trials, including personal stories about clinical trials from patients and researchers
- The UK National Health Service Choices website has information for patients about clinical trials and medical research, including personal stories about participating in clinical trials
- The UK Medical Research Council Clinical Trials Unit also provides information for patients about clinical trials and links to information on clinical trials provided by other organizations
- MedlinePlus has links to further resources on clinical trials (in English and Spanish)

## Introduction

Pharmaceutical clinical trials are increasingly being conducted in the private sector as part of a growing global contract research system [1]–[3]. In the United States, a major shift in the locus of pharmaceutical research has taken place in the last two decades. Whereas the majority of clinical trials were completed in academic medical centers in the 1980s, estimates reported in 2005 showed that more than 70% of US trials were conducted by nonacademic physicians [4]. Community physicians have largely replaced their academic counterparts as principal investigators (PIs) on pharmaceutical trials [1],[5].

US medicine has changed significantly in the past 30 years. As a direct response to the perception that US health care expenditures had reached a point of “crisis,” health care reforms in the 1970s and 1980s aimed to limit excesses in health care spending [17]–[19]. While the reality of this crisis is open to debate, it is a common perception that physicians' payments have decreased significantly due to managed care [20]–[22], whereas practice operational costs have increased [23]. Pham et al. report that US physicians feel “beleaguered” and forced into becoming more “business-oriented” [22]. Physicians have joined larger, multispecialty practices in order to defray costs and liability [24]. Additionally, many have referred patients to their privately owned ancillary service centers [25]. This focus on the business of medicine has raised concerns that medical professionalism in the US, particularly in terms of physicians' values and ethics, has been affected [26].

The pharmaceutical industry has provided an opportunity for US physicians in private practice to serve as PIs on clinical trials [11],[22],[27],[28]. The number of US private-sector physicians conducting pharmaceutical trials increased from 4,000 in 1990 to 20,250 in 2010 [29], with research contracts now worth more than US$11 billion annually [30]. This enormous growth in the number of private-sector physicians involved in contract research is due in part to their replacing academic physicians on pharmaceutical clinical trials, but it is also due to simultaneous exponential growth in the number of clinical trials initiated by industry during this same time period [31].

Pharmaceutical contract research is profitable to US physicians because of the fee-for-service payment system that structures companies' remuneration of PIs [32]. Specifically, beginning with informed consent, PIs get paid for each step in the screening process, and for each study procedure, history and physical, and blood draw. Reimbursement rates are significantly higher than what Medicare pays for the same services [1]. An American College of Physicians' report found that contract research provides individual physicians income of up to US$300,000 annually [33]. Although the potential revenue from contract research is substantial, this income is not without ethical risks. Nonetheless, there is little oversight of financial conflicts of interest in the private sector compared to academic settings, raising concern that if per capita payments are inappropriately high, private-practice physicians might make recruitment decisions that are not in their patients' best interest [34].

Bodenheimer has argued that certain conditions enabled the development of a private-sector clinical trials industry [5]. He explains that, originally, academic medical centers provided (1) trial design expertise, (2) a patient population from which to draw, and (3) prestige and publishing experience. However, the combination of private-sector companies, private-sector physicians, and high-level scientists and physicians employed directly by pharmaceutical companies and contract research organizations has proved a more advantageous drug development model [5]. This model is based on the premise that speeding up clinical development is an important way for companies to increase their profits on patented products and that private companies are more efficient than are academic medical centers [35]. Indeed, it has been reported that private companies complete trials up to three months faster than academic medical centers, resulting in about US$90 million in additional earnings on each new drug [36].

In spite of the dramatic shift in who serves as PIs on industry clinical trials in the US, there has been little research on the implications of this trend [1],[3],[5]. The purpose of our study was to explore the outsourcing of clinical trials to US private-sector research clinics, paying particular attention to the different groups involved in the research enterprise (e.g., pharmaceutical company representatives, physicians, study coordinators, and research participants) and to the everyday practices associated with conducting clinical trials (e.g., recruitment of participants, informed consent processes, and study compliance). The data were collected as part of an institutional ethnography that was conducted between October 2003 and September 2004 [1]. The study was designed in an open-ended way because little empirical research had been conducted on the clinical trials industry and there were few guides indicating specific areas in need of investigation. In June 2011, the authors completed a second-order analysis of the data to use the findings of the empirical project to generate insights about the growing scholarly literature that has cited evidence of an increasing array of unscientific research practices perpetrated by the pharmaceutical industry [3],[4],[6]–[16]. This paper reports on our findings that private-sector PIs view monetary incentives, clinical research, and ethics in unexpected ways.

## Methods

Our study of the clinical trials industry used a qualitative research design, consisting of semi-structured interviews and observation that took place between October 2003 and September 2004 at 25 US private-sector research organizations, such as private practices, dedicated research sites, and large (nonacademic) hospitals. Two cities in the southwest US were selected for the study because of the preponderance of private-sector clinics in this region, according to information in an industry clinical trial clearinghouse database (CenterWatch; http://www.centerwatch.com). The database was also used to identify and contact individual sites. All sites in the two cities were contacted and invited to participate in the project. Although no incentives were offered to promote participation, 75% of sites in one city and 50% of sites in the other city agreed to take part in observation and interviews. According to information listed in the database, there was no discernable difference between sites that agreed or refused to participate, in terms of the types of studies conducted, size of the clinical trials operation, or experience of the site. Clinic specialties included family and internal medicine, gastroenterology, neurology, pediatrics, rheumatology, and urology. As we discuss in more detail below, all sites conducted clinical trials outside of their specialty area. The majority of sites conducted trials to test the efficacy of new products for conditions that already had safe and effective treatments on the market (e.g., allergies, asthma, hypercholesterolemia, and insomnia), as has been identified elsewhere [6]. Only one site consistently tested products for life-threatening conditions (e.g., AIDS or cancer). One site specialized in early phase studies with healthy volunteers.

Semi-structured interviews (Text S1) with 63 informants were clustered to get the perspective of multiple employees at each site, including 12 PIs, 18 coordinators, three recruiters, and seven non-physician site administrators, as well as 14 research participants and nine representatives of pharmaceutical companies. Interviewees were selected based on their availability during observational visits. For instance, during some research visits, the only person present in the clinic was a research coordinator; at other clinics, research visits generated interviews with multiple informants. The size of a clinic and its level of activity limited the number of interviews—and the variety of people available for interviews—at each site. No one who was asked to participate in an interview refused. Interviews were conducted until saturation was reached [37], meaning that additional interviews ceased to provide data that generated new insights or information about contract research. Interviews lasted an average of 40 min. This paper draws on our entire dataset, including themes and findings from observations at all 25 research clinics and all 63 interviews. Because of our focus on physicians in this paper, we quote most extensively from the interviews with 11 physician-investigators who served as PIs in contract research. Physicians in our sample were representative of the national demographics of industry-funded investigators [38]: ten men and one woman; ten white and one Hispanic. Most physicians were in their 40 s, with an age range from mid-30s to late-70s, with a broad range of experience conducting trials (1–20 y).

Observation in clinics was focused primarily on interactions between PIs and participants, as well as between coordinators and participants. Observation ranged from a single visit on one day to multiple visits spanning several months. Field notes were used to capture observational data [39]. In interviews, informants were asked questions about their job responsibilities as well as about their experiences working in clinical research, how research had changed over time, and what types of changes they would like to see in the future. Specific interview questions for PIs also solicited their perceptions of research ethics and of their responsibilities to participants, pharmaceutical companies, and science. All data were collected by one of the authors (J. A. F.), who is trained in qualitative sociological methods. This author also transcribed all the interviews in full and contacted interviewees to give them the opportunity to edit their transcripts. The study was reviewed and approved by the Institutional Review Board of Rensselaer Polytechnic Institute. Written informed consent was obtained from all participants. All research clinics and informants were given confidentiality as part of their participation in the study.

In keeping with a methodology of grounded theory [40], there were no predefined theoretical or conceptual frameworks or specific hypotheses identified for analysis prior to data collection. In addition, multiple conceptual frameworks were developed through the process of interpreting the findings from the project. As with most grounded theory projects, the data analysis relied on a multi-staged process of coding field notes and interviews in their entirety for core and emerging categories. All the coding was done by one of the authors (J. A. F.). Coding was multi-staged in order to revisit the data multiple times for depth of analysis and for the creation of cross-references among the data and the categories coded. Coding during the data collection period was done by creating detailed memos at the conclusion of site visits and by repeated, fine-grained reading of transcripts and field notes for additional themes that emerged as important. This process helped to guide subsequent observation and interview techniques by highlighting areas of inquiry that needed further exploration. In the final stage of coding after the data collection period, more subtle codes were added that aimed to create subcategories within emergent themes. The current analysis was validated in a 2-fold way by both authors: first, by reflecting on themes in light of the published literature on the topic and, second, by attending industry conferences and reading industry publications designed to provide advice and commentary on the clinical trials industry.

## Results

Our research revealed trends in why US physicians become PIs on industry clinical trials and how they develop their professional identities as contract researchers. By “professional identities” we are referring to how physicians incorporate into their existing clinical roles their experience of the rights and responsibilities that accompany contract research. We discuss three aspects of their identities shaped by (1) their motivations to conduct clinical trials, (2) an orientation that privileges business over science, and (3) an industry-based sense of research ethics.

### Motivations

All private-sector physicians we interviewed reported the financial incentives of participating in pharmaceutical trials as the most salient for becoming contract researchers. The dominant view among all our informants—physicians, research staff, and pharmaceutical company representatives alike—is that contract research is a lucrative activity to integrate into a clinical practice. One physician said:

We're paid less and less for the work that we do. In fact, we're paid about 50% of what we made 20 years ago…. So one of the ways that we have to try to offset that [trend] is to find an alternative source of revenue; getting into research was a way to do that.

Not only is contract research viewed as more profitable than standard care, but the physicians also viewed it as a way to shorten their hours in the clinic because many study activities can be delegated to nurse coordinators. An administrator in charge of recruiting additional private-practice physicians as PIs for a large national company explained:

It's a profitable industry. Doctors want to do the research because it's profitable. They don't want to spend the time to do it, and right now, the way the industry's set up, they can do that. They can hire a nurse and a couple coordinators to essentially run 98% of the study for them, make them a lot of money, and they do minimal work.

Indeed, several study coordinators in our sample joked about how clinical research helped physicians improve their golf games (*n* = 8, 44%). Our observations confirm that at the majority of the 25 sites at which we conducted our research (*n* = 18, 72%), the PIs were frequently absent from the clinic.

Financial incentive was not the only reason physicians in our sample gave for becoming contract researchers. The physicians also noted that clinical research is intellectually rewarding and elevates their professional status. In addition, almost half of the physicians (*n* = 5, 45%) found clinical trials to be a benefit to their patients or the broader community. One physician said:

What we do here is I can give out free blood pressure, diabetes, and lipid medication as well as free blood pressure, diabetes, and lipid *care* to the community…. A lot of my patients come from a lower socioeconomic background, and so it's very nice because I sort of had the ideal when I went into medicine that I wanted to do that, but somehow you get taken away from that as years go by.

In spite of these other perceived benefits of becoming an investigator, our data show that financial gain tends to be the *initial* motivation for physicians to seek a study contract.

### Business versus Science

Our research revealed that PIs perceive their roles in terms of business rather than science. In fact, because of the rapid growth of this industry, many physicians in our sample (*n* = 9, 82%) asserted that PIs must be competent businesspeople in order to succeed economically in contract research. As one physician explained:

There's a lot of business in the industry. And medical training is certainly important because there's, of course, a lot of medicine in there; but that's not enough, it's not sufficient. Clinical trials is a specialty unto itself, both the business of clinical trials as well as the ethics of clinical trials, dealing with IRBs [institutional review boards], dealing with pharmaceutical companies, dealing with a whole bunch of things, as well as how to manage a large [research] center.

As evidence of this professional identity, our interviewees differentiated themselves from their academic counterparts by their participation in different conferences and sessions to fulfill their continuing medical education requirements: private-sector PIs often attend business-related panels whereas academic PIs tend to participate in panels devoted to science and clinical advances. One physician said:

I go religiously to the [annual specialty meeting] strictly for business development purposes…. That doesn't mean that I don't like and appreciate the science…. I try to understand the direction each pharmaceutical company goes when they're using this compound in this indication, which is targeting blah blah blah, whatever it is.

While conference session attendance is only one small part of physicians' professional identities, our interviewees also claimed that investigator competency is not based on clinical expertise but on savvy in managing trials. Private-sector PIs believe that they offer something to the pharmaceutical industry that academics do not—the ability to carry out a diverse range of trials more quickly and effectively—regardless of their medical specialty. One physician explicitly compared the expertise of private-sector trialists and academic physicians:

What do we bring that the academic medical centers don't? We bring a *techné—*it's an old Greek word—as opposed to a science. Pharmaceutical companies can hire the scientists, but at some point the end has to be executed, so that's what we bring to the table: our capability, interest and motivation, drive. Because that's it for most of us—not all, but most of us—in the private sector, it is a priority for us to succeed…. What [we] offer is an execution.

Moreover, none of the PIs in our sample ever participated in analyzing trial data or authoring publications.

In our sample of research organizations, we also found that private-sector PIs are involved in a broad range of therapeutic areas. Because the focus is on “execution” rather than clinical expertise per se, private-sector physicians routinely served as PIs on clinical trials for diseases outside of their specialty training (*n* = 9, 82%). One physician—who was trained as a neurologist but had ongoing studies for depression, weight loss, and gastroesophageal reflux disease, among others—explained his approach:

I do not do original research; I do *contract* research. I read the neurology literature; I do not read the psychiatry literature; I don't read the obesity literature. You know, for certain things, I'm pretty smart. And in other things, I don't even pretend that I'm smart about those. But I'm *very* good at running clinical trials, so in a sense…I'm a highly skilled *practitioner* of clinical trials who has an appreciation for the science, plus the scientific need, plus the fact that they rely on the data that we give them to be scientifically valid because there are lives at stake in that data. But I'm *not* a scientist.

The predominant perception we found among our informants—including representatives from pharmaceutical companies—is that skilled contract researchers can conduct any clinical trial provided that they can recruit the relevant patient populations into those studies.

### Ethics

We found that reframing clinical trials as a business endeavor is also associated with physicians' perceptions of research ethics. When asked about “ethical” issues that come up in clinical trials, physicians in our sample focused instead on responsible conduct of research. For example, one physician explained that the PI's responsibility is to be “very conscientious, and they also do everything ethically based on the protocol, regulations, FDA [US Food and Drug Administration], etc.” In other words, PIs in our sample answered by talking about the importance of following the protocols and avoiding misconduct or fraud. Surprisingly, physicians had a lot to say about the temptations of “massaging the data,” as this statement by an experienced PI illustrates:

There's all sorts of ways people will justify blurring lines of distinction, which may or may not be clear actually. Throwing away a lab value is way over the line, right? Does it have to be fudged when you're doing a blood pressure study and this person is two points out of range on their fifth visit, and you've already put in a month of time on that person? I don't know. Does that betray the spirit of what you're trying to do? As opposed to ten points out of line, then they're out. So I can see how individual people will sort of figure out where they're comfortable on that.

According to another physician:

Obviously, there's an ethical standard that the physicians will be following, but, of course, everyone knows there's shades of pressure around your proper ethical behavior which may be trying to push or negotiate certain decisions that the physician is making or certain opinions that they're forming, to comply or go along better with others [at sponsoring companies] who are pressuring him or her [to enroll/retain participants].

What was striking in our research was that more traditional views of ethics, especially the investigator's responsibility to trial participants, were not explicitly evident. In part, this might be due to the prepackaged nature of the contract research enterprise, but it might also be due to private-sector physicians' lack of scientific training and their trust in pharmaceutical companies. For example, one physician explained:

You know, we really don't have a lot of leeway in the scientific department. I mean, if somebody says we have this really great drug that works for blood pressure, I have no idea how this damn thing works! But I'm still going to go down the hall and do physicals and check blood pressures and sign off adverse events…. I have no idea about science! Whether that's good or bad or indifferent. That's why we're doing the research, and I figure someone's putting several million dollars, or $20 million into doing this study, so they must believe in what they're doing.

In other words, for this physician, the amount of money that pharmaceutical companies invest in the development of their products becomes the basis of trust that these companies are making scientifically sound and ethical decisions in the design of clinical trials.

## Discussion

The results from our study corroborate earlier research indicating that US private-sector physicians are motivated to participate as PIs in pharmaceutical clinical trials for financial reasons [5],[11],[22],[32]. We also discovered that while financial incentives might be the most salient, at least initially, physicians also report other advantages associated with contract research: intellectual reward, increased professional status, and benefit to their patients and communities.

Beyond private-sector physicians' motivations to participate in pharmaceutical trials, our findings reveal that the financial aspects of contract research are aligned with their professional identities. Instead of seeing themselves as scientists or researchers, they see themselves as trial practitioners and as businesspeople. At conferences the physicians in our sample attend sessions geared toward the business aspect of trials, suggesting that they might not consider additional knowledge about pharmaceutical science or medical practice to be of foremost importance for their professional development. This may help to explain the rise of industry conferences that are entirely dedicated to specific business aspects of trials, including patient recruitment [41]. Additionally, it was an unexpected finding that the financial incentive to participate in contract research propelled the private-sector physicians in our sample to serve as investigators on trials for investigational drugs in a broad range of therapeutic areas. That they often work on the margins of their specialized training provides one potential explanation for why the private-sector physicians did not see themselves as scientists and claimed little insight into the mechanisms by which particular drugs work.

Unlike traditional investigator-initiated research, such as studies funded by US National Institutes of Health, the prepackaged nature of contract research means that physicians do not need to have expertise in research design and methods because they have limited input into trial protocols [1]. As a result, private-sector investigators, who by self-report in our study are not interested in or trained to understand the science, potentially facilitate the pharmaceutical industry's ability to exert more control over information about their products, including any negative data generated from clinical trials [9],[12],[13],[42]–[47]. The literature has suggested that pharmaceutical companies are interested in controlling their trial datasets so that individual PIs do not have access to data collected at all participating investigative sites and any resulting publications can be prepared by ghostwriters [6],[10],[16]. Our findings indicate that this arrangement tends to be acceptable to private-sector physicians who rarely—if ever—participate in analyzing trial data or authoring publications.

Private-sector physicians incorporate ethics into their identities in unique ways as well. It was revealing that when asked about ethics, the physicians in our sample always answered the question in terms of responsible conduct of research. Instead of being concerned with the dominant domain of research ethics—the PI's responsibilities to research participants—they understood their primary “ethical” responsibility as providing accurate data to the companies that hired them. Within the context of their work, this makes sense. Contract researchers do not design the studies, nor are they given much opportunity to provide input on the protocols. Additionally, private-sector physicians often do not have institutional review boards associated with their clinics, and the review of clinical trial protocols is handled by centralized commercial companies [8]. These two factors imply that to a large extent PIs must trust that the sponsoring company has designed a scientifically valid and ethical trial and that the centralized institutional review board has established appropriate guidelines for the protection of enrolled research participants [27]. What this suggests, however, is that physicians do not perceive the potential ethical conflicts that emerge as a result of their dual roles as health care provider and clinical researcher when they are conducting clinical trials that enroll their own patients.

There are, of course, limitations to our study. Although we included a diverse sample of research organizations in our study and reached thematic saturation with our interviews, the number of physicians we interviewed was small. We limited our study to private-sector research in one geographical region of the US. While the published literature indicates that the trends we found in our research are likely generalizable to other regions of the US, the context differs dramatically in countries with national health care systems, so it is likely that private-sector PIs characterize their roles differently in other settings. We must also note that our data were collected eight years ago, so it is unclear how physicians' identities might have changed in response to subsequent changes in the funding of contract research, such as the rise in seeding trials, or the broader health care environment. In addition, our study was not designed to investigate physicians' professional identities as contract researchers. Instead, what we report here are unprompted themes identified during data analysis. To better understand private-sector PIs, future studies are needed to compare their identities with those of academic physicians and to investigate how differences in identity might influence the execution and results of contract research. Future studies could also compare cross-national differences in the perceptions and practices of pharmaceutical investigators. Indeed, contract research is a global enterprise [2], and our study highlights the need to understand better the changes that have occurred in the conduct of pharmaceutical clinical trials worldwide.

### Conclusions

The focus of this paper has been on the participation and professional identities of US private-sector physicians who conduct pharmaceutical clinical trials. While a greater number of physicians in more diverse settings are now engaged in pharmaceutical research than were 20 years ago, our data have potentially troubling implications for drug development. We found that the private-sector physicians interviewed identify primarily with the business rather than the science of contract research. In addition, our findings indicate that the private-sector PIs aligned their sense of research ethics with industry—ensuring the responsible conduct of research—rather than foregrounding the interests of research participants. Because these private-sector PIs are largely motivated by financial gain as opposed to making a contribution to science, we suggest that the professional identity of private-sector PIs may inadvertently offer pharmaceutical companies the ability to exert more control over their proprietary information and clinical trial data. This provides one explanation as to why, despite the participation of physicians as PIs, pharmaceutical companies have been able to suppress negative trial data and selectively publish study results [9],[12],[13],[42]–[45].

We do not question that contract researchers are able to execute studies professionally and effectively, even when they are business-oriented. That said, private-sector physicians are an important part of a different type of contract research system, one that has become the dominant model of conducting pharmaceutical clinical trials in the US and around the world [1]–[3],[48]. This change can be thought of as facilitating a shift in drug development in which protocols are developed and study results are analyzed in-house by pharmaceutical companies—sometimes in conjunction with their marketing departments [6],[7]. We suggest that the lack of interest or expertise in the science of clinical research reported by private-sector PIs in our study would facilitate a research enterprise that is characterized by high levels of industry control over research protocols, data analysis, and dissemination of information about new pharmaceuticals.

## Supporting Information

Text S1 Interview guides.
(DOC)

## Abbreviations

PI: principal investigator
