# Supplementary material for: United States Private-Sector Physicians and Pharmaceutical Contract Research: A Qualitative Study
Source: PLoS Med. 2012 Jul 24;9(7):e1001271. doi: 10.1371/journal.pmed.1001271 (PMC3404112; doi:10.1371/journal.pmed.1001271)
Supplement: Text S1 — Interview guides. (DOC) [file pmed.1001271.s001.doc]

Text S1. SEMI-STRUCTURED INTERVIEW GUIDES

*Investigators/Research Staff*

- Please describe the clinical trials on which you are currently working.
- Where do you receive funding from for this research?
- Are any private companies involved in the research protocol? What, if any, labor is outsourced to CROs, SMOs, recruitment companies, etc.? Who is overseeing your work? Whose work do you oversee?
- What are the current strategies used to recruit volunteers for this research? How were these strategies decided upon? How effective do you perceive them to be? If budgetary constraints were not present, how might you recruit volunteers differently? How necessary do you think are third party recruitment companies in successfully enrolling volunteers?
- What are the personal benefits that volunteers can derive from this research? What are the incentives volunteers receive for participating in the study? How were these incentives decided upon? Why are they necessary? Do you think these are adequate benefits in exchange for their participation? Why or why not?
- Are there any financial incentives for your participation in the research? Please describe these. What professional incentives do you have for your participation in the research? What financial incentives do you have for recruiting volunteers into the study? What do you have to gain from the success of this study? What do you have to lose if the study fails?
- How do you think these incentives influence your judgment during screening and enrollment? What are your concerns about the recruitment practices used in this study? Do you consider yourself to have any conflicts of interest within this research protocol? What are they? Why do these conflicts of interest appear? How do you identify possible conflicts of interest that arise during the study that may compromise your role or the study itself? How do you deal with these conflicts of interest?
- What is the process of informed consent for this particular research protocol? Do you feel it is adequate in informing potential volunteers? How much do you think the informed consent process has an impact on volunteers’ decision to participate in the research? How might informed consent be improved?

*Human Subject Volunteers*

- How long have you been aware of the possibility of participating in medical research? Have you done it before? Have you considered it for a while? What made you interested in this particular study? How and when did you find out about this study? Why do you think you are eligible for participating in this study?
- Have you decided to participate in this study? When did you decide to participate in this study?
- Tell me about the research that you will take part in. What are the benefits for you? What are the risks to you? Why do you think you are eligible for this study? What did you have to do to be able to take part in this study?
- What does informed consent mean? What was the process of informed consent that you went through? Did you have questions that you wanted to ask about the research? Did you ask any of them? Did you feel that you were allowed to ask questions? Why or why not? What do you think would have been the investigator’s reaction if you had decided not to participate? Did this influence your decision?
